# Improving effectiveness of online learning for higher education students during the COVID-19 pandemic

**DOI:** 10.3389/fpsyg.2022.1111028

**Published:** 2023-01-16

**Authors:** Xuelan Li, Zhiqiang Pei

**Affiliations:** School of Management, Anhui Science and Technology University, Bengbu, Anhui, China

**Keywords:** online learning community, group dynamics theory, cluster analysis, analytic hierarchy process, COVID-19

## Abstract

During the COVID-19 pandemic, online learning has become one of the important ways of higher education because it is not confined by time and place. How to ensure the effectiveness of online learning has become the focus of education research, and the role of the “online learning community” cannot be ignored. In the context of the Internet of Things (IoT), we try to build up a new online learning community model: (1) First, we introduce the Kolb learning style theory to identify different online learning styles; (2) Second, we use a clustering algorithm to identify the nature of different learning style groups; and (3) Third, we introduce the group dynamics theory to design the dimensions of the questionnaire and combine the Analytic Hierarchy Process (AHP) method to identify the key influencing factors of the online learning community. We take business administration majors and students in universities as an example. The results show that (1) as a machine learning method, the clustering algorithm method is superior to the random construction method in identifying different learning style groups, and (2) our method can well judge the importance of each factor based on hierarchical analysis and clarify the different roles of factors in the process of knowledge transfer. This study can provide a useful reference for the sustainable development of online learning in higher education.

## 1. Introduction

During the COVID-19 pandemic, online learning has become the only alternative to traditional teaching methods in higher education. However, online learning lacks timely face-to-face feedback and interaction, and it is difficult to establish close links between individuals. Therefore, ensuring the efficiency of online learning has become a mainstream topic in the research field of education. This is closely related to online learning communities, student awareness, and technological developments. Let us start with a review of the relevant background. The continuous innovation of information technology has greatly affected people’s learning, work, and life style. Online education, because of its convenient and open characteristics, is increasingly changing learning behaviors. In 2019, China’s online education market grew by 21.47% year on year, reaching 346.8 billion yuan (data from CNNIC); in 2020, the Ministry of Education issued the Guiding Opinions on Doing a Good Job in the Organization and Management of Online Teaching in Colleges and Universities during the Epidemic Prevention and Control Period and put forward the slogan of “teaching and learning without stopping.” Online learning has officially become an irreplaceable way for teachers and students to share classes. The online learning community is a major component of the online education system. It uses social networks as a carrier to establish learning cooperation relationships among participants to exchange experiences, share resources, and pursue the common development of individuals and groups. However, in educational practice, there are a large number of “invisible participants” and “marginal participants” in the online learning community who lack learning enthusiasm and initiative and have a negative impact on the effectiveness of online learning. It is an urgent problem for higher education to excavate the influencing factors of the efficiency improvement of the online learning community, improve the participation of learners, and promote the long-term development of the online learning community.

Business administration is based on the basic theories of management and economics and the use of modern management methods and means for effective enterprise management and management decisions. Business administration is a major widely offered by universities all over the world. With the acceleration of the process of global economic integration and the deepening of the economic system reform of various countries, a large number of business administration talents with modern management concepts and skills are needed, which provides a very broad prospect for the development of students in this major. In the new era, colleges and universities pay attention to training students’ practical ability, social adaptability, and job competitiveness. Students majoring in business administration generally reflect the characteristics of active thinking, an outgoing personality, good communication, and so on. The career orientation also puts forward higher requirements for the comprehensive quality of the major. Therefore, this article takes the major of business administration as the research object, through learning style and cluster analysis, independently constructing the online learning community, and explores the influencing factors of its development based on the theory of group dynamics, then proposes targeted online learning community development strategies so as to dynamically combine the structural level design with the strategic level adjustment and promote the online learning efficiency of professional courses in universities.

## 2. Literature review

During the COVID-19 epidemics, how to carry out effective online learning and how to improve the efficiency of online learning in universities have become hot research topics.

1. From the perspective of online and offline integration in higher education, the role of educational technologies in the transition from face-to-face to online teaching and learning activities and five challenges to transitioning to online education experienced by higher education institutions such as synchronous/asynchronous learning tool integration, access to technology, faculty and student online competence, academic dishonesty, and privacy and confidentiality, were identified (Turnbull et al., 2021). The problems, challenges, and advantages of using e-learning systems instead of traditional education in higher education were revealed, and surveys and empirical tests on teachers and students at the University of Benghazi were conducted (Maatuk et al., 2022). Five trends affecting online learning were summarized as follows: the intersection of different learning modes; super-large-scale popular learning; the openness of education and political game; the interaction between students, teachers and students, and students and content; and the diversification of digital technologies (Mark et al., 2022).
2. From the perspective of the online learning environment, as the actual presentation becomes challenging, the online learning environment puts higher demands on teachers, forcing them to develop and master information technology as soon as possible and expand the practical activities in the fields they teach (Mitchell, 2020). Kohnke and Moorhouse (2020) pointed out that tools and resources were important components of an online learning environment. The real-time exchange of information in online learning was usually carried out through video conferencing tools such as Zoom and Skype. Reddy et al. (2021) constructed an online learning framework and learning intervention module composed of media, information, and technology (computer, visual, and communication literacy) according to the challenges posed by digital literacy enhancement. Information and communication technology (ICT) is the tool base for online learning and provides individuals and organizations with the creativity that can facilitate innovative online education environments (Abdullah et al., 2019; Altawaty et al., 2020).
3. From the perspective of the role and behavior of online learning subjects, Kim et al. (2020) divided online learners into transformational leaders and exchange leaders. Song et al. (2022) obtained the original index data by using the online teaching platform with independent intellectual property rights, analyzed and summarized the characteristics of the online learning behavior of specific groups, which provided a targeted basis for improving the knowledge mastery level and learning interest of college students, and improved the teaching quality. The influencing factors impacting university students’ behavior and intentions to use social media were figured out to improve their academic performance during the COVID-19 pandemic (Alismaiel et al., 2022). Cheng et al. (2020) found qualified online collaborative learning leaders were better at promoting the skill development of collaborators. Haataja’s research found that monitoring could promote effective interactions among collaborators and continuously advance their collaborative learning tasks (Haataja et al., 2021). Peng and Wang (2021) explored the dynamic evolution of hybrid online learning and the relationship between participants by using structural analysis. Ma and Gu (2008) summarized the characteristics of teachers in the online learning community. Wang (2018) studied the influence of leading nodes in the online learning interaction network based on the theoretical model of online learning interaction between teachers and students.
4. From the perspective of effect evaluation and improvement strategy of online learning, Khosravi et al. (2021) proposed an artificial intelligence intervention scheme to help educators identify, explore, and select appropriate intervention measures based on online analytical processing, data, and process mining technologies. A model based on a machine learning algorithm was proposed to prevent academic risk, and its supervisory effect was verified in the multidimensional academic performance of more than 2000 college learners (Karalar et al., 2021). The construction of an online learning community is one of the important ways to improve the efficiency of online learning in colleges and universities. Under the influence of COVID-19, online teaching should be student-centered, form a process feedback mechanism, strengthen the support of information technology, and build a multi-dimensional teaching evaluation system. (Li and Liang, 2022). Li and Wang (2018) constructed the social system structure of the online learning community from four perspectives, namely, team organization, collaboration and interaction, knowledge innovation, and knowledge generation, and proposed four dimensions of organizational strategies, namely, team organization, interactive communication, self-reference, and self-production. Tao (2018) explored the construction of an online learning community from the three dimensions of prediction, migration, and enhancement mechanisms based on social learning theory. Zhao and Yan (2013) analyzed the influencing factors of in-depth interaction among members of the online learning community based on the formation of the community and the degree of interaction between participants. Elaine and Michael (2015) evaluated the learning effectiveness of the online learning community from the three perspectives of intellectual, social, and emotional transformation. Wang and Gu (2020) constructed the dynamic development mechanism model of the online learning community from the three perspectives of cohesion, driving force, and security forces to promote the operation quality of online classrooms. Liu (2020) explored the promotion of online collaborative learning quality from the perspective of emotional interaction among members of the online learning community.

It can be seen that the academic circle has a lot of research on online learning in colleges and universities in the context of the epidemic, but most of them are single-level discussions on online learning methods or promotion strategies, which are likely to lead to the overall effect of structural level (improvement of learning methods) and strategic level (realization of learning objectives and promotion strategies) not meeting expectations. Making comprehensive research on improving the effectiveness of online learning for higher education students during the COVID-19 pandemic is a topic both universal and important. This article tries to answer the following questions:

1. How to design and construct effective online learning methods?
2. How to judge the influencing factors of effective online learning methods?
3. How to find the different importance of each influencing factor to improve the efficiency of online learning in colleges and universities?
4. How to promote implementation strategies of effective online learning methods?

This study is divided into five sections. In addition to Section 1, Section 2 states the literature review; Section 3 describes the theoretical basis, research methods, and data sources, Section 4 gives the results, and Section 5 discusses the conclusion and policy implications.

## 3. Theoretical basis, research methods, and data sources

### 3.1. Theoretical basis

#### 3.1.1. Group dynamics

Lewin, the founder of topology psychology, put forward “group dynamics” in 1939, which is also called “group mechanics.” It is a theoretical framework to describe various elements and their relationships in a group. It focuses on the interaction and influence between different forces in a group, which can be summarized into three dimensions, namely, group solidarity, group development, and group support. Among them, group solidarity is a concentrated reflection of the interaction and close relationship of various elements within a group, which reflects the level of group cohesion and the level of individual trust in the group. There are usually three sources: material ties, emotional ties, and ideological ties. Group development is the process of self-optimization of a group in the process of movement, and the result of positive interaction between groups and individuals, and individuals and individuals. Group goals and group motivation are the direct driving forces to promote group development, which determines whether individuals have clear and consistent goals and are willing to make efforts to achieve them. Group support reflects the structural characteristics of a group and its ability to support the group. It respects the differences of members, optimizes the combination of members, helps to improve the consistency of the wishes and actions of different members, and promotes close cooperation and efficiency. To explore the development strategy of the online learning community, we must start from the beginning. Taking the group dynamics theory as the starting point, starting from the three dimensions of group solidarity, group development, and group support, we can investigate the group behavior, fully consider the mechanism of each element, find out the key factors to optimize the efficiency of *the* online learning community, and give targeted suggestions.

#### 3.1.2. Learning style models

**Figure 1:**
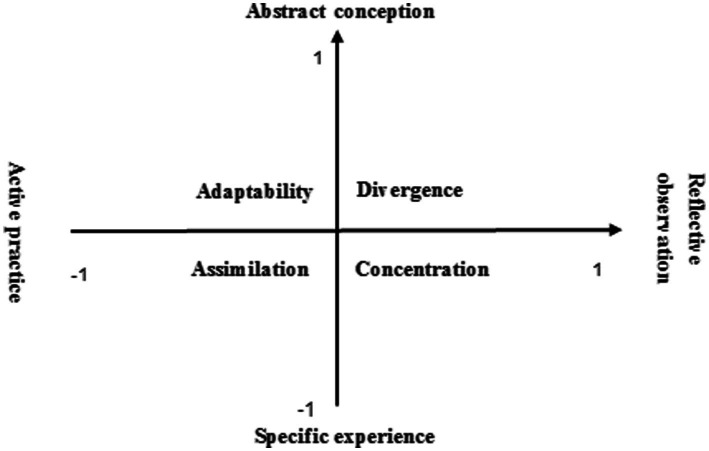
Kolb learning style model.

Educator David Kolb put forward the learning style model in 1976. He believed that there were differences in the way each person received learning information in the process of learning activities, which led to the formation of a unique learning style, mainly embodied in two aspects, namely, perception and information processing. The dimension of perception mode includes two directions, namely, “collective experience” and “abstract concept” (concrete perception and abstract perception), which reflects the individual’s preference *for* understanding the environment and acquiring experience. The dimension of information processing includes two directions, namely, “subject practice” and “reflective observation” (two kinds of processing activities, i.e., positive action and reflection summary), which reflects the differences in the way individuals process or transform information when receiving information. Combining these two dimensions, we can get a model that reflects the individual learning style, generating four types of learning styles, namely, divergent, assimilative, centralized, and adaptive (as shown in Figure 1).

### 3.2. Research methods

#### 3.2.1. Cluster analysis

Learners are the center of the online learning community. Defining learners’ learning styles can meet the differentiated requirements in learning cooperation and interaction, give play to the advantages of members with different styles, promote the construction of *a* common knowledge system for learners, improve the depth and breadth of thinking about the learned knowledge, and ensure the operation*al* efficiency and long-term development of the online learning community. According to Kolb’s learning style model, establish two-dimensional coordinates and formulas S = {(Sp, Sq)|Sp, Sq∈[−1, 1]}, vector S represents learning style, Sp and Sq, respectively, represent learners’ preference in information processing and perception dimensions. The greater the absolute value is compared with 0, the higher the tendency degree is, and positive and negative values represent the tendency style, such as subject practice/reflective observation. The Kolb Learning Style Questionnaire is used to investigate learners, determine the preference of each member (Sp and Sq), and use hierarchical clustering to cluster members with similar learning styles. Hierarchical clustering methods can be divided into two categories, namely, agglomeration and fragmentation. In this *article*, *the* agglomeration clustering method is used. In a bottom-up order, first, assume that all members are grouped separately. According to the similarity of each member’s learning style (usually the closest two), merge the two clusters. Repeat the above steps until all clusters are merged into one cluster to form a pedigree. The clustering is reasonably divided to form n online learning communities. At this time, the learning styles of learners in each online learning community are very similar, showing homogeneity within groups and heterogeneity between groups, which does not conform to Kolb’s learning style model. Select the cluster with the largest number, select K learners in turn to form K online learning communities, and select one member from other clusters to merge until all members are merged. At this time, the cluster that has formed meets the requirements of intragroup heterogeneity and intergroup homogeneity and is the ultimate online learning community.

#### 3.2.2. Analytic hierarchy process

The analytic hierarchy process (AHP) is a decision-making method that decomposes the elements always related to the decision into levels such as goals, criteria, and schemes and then carries out qualitative and quantitative analysis on this basis. Using this method to make an analysis of the factors influencing the development of an online learning community based on group dynamics.

### 3.3. Data sources

#### 3.3.1. Questionnaire design

The essence of exploring the influencing factors for the development of an online learning community is to explore the role of various elements in the development of a learning community through the online knowledge transfer process, including the interaction between the whole and the individual and the role between individuals. Based on the group dynamics theory, the questionnaire was designed, and the development of an online learning community was taken as the target layer to establish an indicator system. Three dimensions and 17 indicators were designed. The questionnaire is divided into two parts. The first part involves the gender, grade, and basic use of the online learning platforms of the interviewees; the second part is the main part of the questionnaire, which investigates the influencing factors from the perspectives of group solidarity, group development, and group support. The questionnaire is divided into 1–5 levels according to the importance by Likert scale, of which Level 1 is the least important and Level 5 is the most important. The interviewees score each indicator factor. The specific questions of the questionnaire are shown in Table 1.

**Table 1:** The hierarchical model of influencing factors.

**Target layer**	**Criterion layer**	**Scheme layer**	**Indicator description**
Development of online communities	Group solidarity T1	T11 Common Goals	Have clear group learning objectives
		T12 Post and communicate	Number of times members post and communicate in the discussion area
		T13 Resource sharing	Members are willing to share learning resources
		T14 Group norms	Members’ awareness and compliance with group norms
		T15 Collective credit	Members have a sense of collective honor
	Group development T2	T21 Personal planning	Members have clear learning plans
		T22 Access frequency	How often members visit the online learning platform
		T23 Online duration	The duration of a member’s single visit to the online learning platform
		T24 Resource acquisition	Number of times members download online resources
		T25 Information processing	Ability to collect and screen information and build knowledge
		T26 Review summary	Experience summary and condensation in the learning process
	Group support T3	T31 Division of tasks	Reasonable division of labor within the community
		T32 Clear positioning	Members are clear about their professional development orientation
		T33 Resource provision	Coverage of online resources
		T34 Typical character	The exemplary role of typical figures in the community
		T35 Teacher guidance	The guiding ability and interactive efficiency of professional teachers
		T36 Supervision and monitoring	The supervision of the teaching process by the university and college supervisors

#### 3.3.2. Questionnaire distribution and recovery

The questionnaire was distributed among the students majoring in business administration in Anhui Science and Technology University through the questionnaire star. A total of 281 questionnaires were collected, including 247 valid ones, with 93 male students and 154 female students. There were 83 senior students, 64 junior students, 59 sophomores, and 41 freshmen.

## 4. Results

### 4.1. Example of construction of online learning community

The business administration major of Anhui Science and Technology University is selected as the experimental class. Taking Class 182 of business administration as an example, 28 students are numbered A1-A28 in turn. Their learning style preferences are shown in Table 2.

**Table 2:** Learning style preference.

Number	Information processing dimension	Perceptual dimension	Number	Information processing dimension	Perceptual dimension
A1	0.33	−0.69	A15	0.23	−0.62
A2	−0.33	0.45	A16	−0.49	0.6
A3	0.18	−0.66	A17	−0.39	−0.45
A4	−0.42	0.63	A18	0.87	0.36
A5	−0.45	−0.42	A19	−0.51	−0.48
A6	0.93	0.33	A20	0.64	0.46
A7	−0.42	−0.45	A21	0.85	0.76
A8	0.72	0.42	A22	−0.75	0.53
A9	0.9	0.87	A23	0.25	−0.72
A10	−0.72	0.69	A24	−0.43	−0.5
A11	0.21	−0.78	A25	0.47	−0.62
A12	−0.39	−0.54	A26	−0.28	0.84
A13	0.36	−0.43	A27	−0.35	−0.71
A14	−0.41	0.5	A28	0.46	0.68

**Figure 2:**
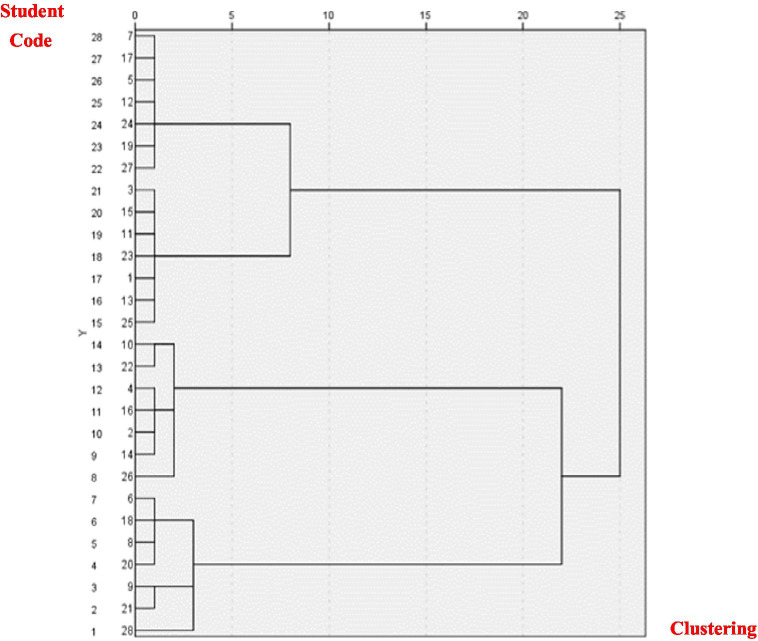
Cluster pedigree diagram.

The SPSS software is used to cluster the data, and the results are shown in Figure 2. Four online learning communities (*K* = 4) are preliminarily constructed. According to the pedigree diagram, the online learning community was preliminarily constructed, and C1: A7, A17, A5, A12, A24, A19, A27 were obtained; C2: A3, A15, A11, A23, A1, A13, A25; C3: A10, A22, A4, A16, A2, A14, A26; C4: A6, A18, A8, A20, A9, A21, A28 and other four online learning communities, at this time, the number of clusters is equal and the internal homogeneity, *thus,* any cluster can be combined. Here, take C1 (the number of clusters is equal, which can be taken arbitrarily) to generate seven new clusters: C11: A7, C12: A17, C13: A5, C14: A12, C15: A24, C16: A19, C17: A27, one learner is selected from other clusters for merging, and finally seven learning communities are formed. The results are shown in Table 3. In cluster construction, the number of members in the online learning community is the same, and the average style vector (i.e., the average value of the group’s learners’ preferences on the two dimensions) is close, indicating that the construction method ensures homogeneity between groups. At the same time, comparing the learning styles of each member of the community, there are significant differences, indicating that there is heterogeneity within the group. In the random construction method, the number of community members varies greatly, and the overall average style does not fully control the homogeneity. There are no significant differences among the members of the community, and their learning styles are relatively similar.

**Table 3:** Comparison of community construction results.

	**Cluster construction**			**Random construction**	
**Community**	**Member**	**Average style vector**	**Community**	**Member**	**Average style vector**
C11	A7,A3,A10,A6	(0.118, 0.032)	C11	A9,A21	(0.09, 0.07)
C12	A17,A15,A22,A18	(−0.007, −0.22)	C12	A2,A4,A14,A16,A26	(−0.39, 0.60)
C13	A5,A11,A4,A8	(0.015, −0.038)	C13	A1,A3,A11,A13,A15,A23,A25	(0.29, −0.65)
C14	A12,A23,A16,A20	(0.0025, −0.05)	C14	A10,A22	(−0.74, 0.61)
C15	A24,A1,A2,A9	(0.072, 0.088)	C15	A5,A7,A12,A17,A19,A24,A27	(0.42, 0.51)
C16	A19,A13,A14,A21	(−0.01, −0.045)	C16	A6,A8,A18	(0.84, 0.37)
C17	A27,A25,A26,A28	(0.075, 0.048)	C17	A20,A28	(0.55, 0.57)

### 4.2. Reliability and validity analysis of the questionnaire

SPSS22.0 was used to evaluate the reliability of the questionnaire, and the results are shown in Table 4. Cronbach’s α is an indicator used to evaluate the reliability of the questionnaire. If the indicator exceeds 0.9, it indicates that the reliability of the questionnaire is high. According to the analysis results, Cronbach’s α value is 0.961 > 0.9, indicating that the research questionnaire has high reliability and can be used; All CITC values were above 0.4, indicating a high degree of correlation between them. The KMO value calculated by the maximum variance method is 0.912, which is greater than 0.6, indicating that the validity of the data is high. The data passed the Bartlett spherical test. After analyzing the interpretation rate of the rotated cumulative variance, the calculated value is 60.47% > 50%, which indicates that the information o*n* each research item can be effectively used. The common factor variance of all items was greater than 0.4. Except for T11, T31, and T35, according to the value of *the* factor load coefficient, the classification and corresponding factors of each item are consistent with the expected dimensions. According to the analysis results, T11 and T31 w*ere* included in factor 1, which is jointly named “group development” with T21–T26; T12–T15 was included in factor 2, named “group solidarity”; T32–T35 was included in factor 3 and named as “group support.” The classification of item T36 has the phenomenon of “confusing one thing with another”, thus, this item is excluded (see Table 5).

**Table 4:** Reliability analysis.

**Index name**	**Correction item total correlation (CITC)**	**Item deleted α coefficient**	**Cronbach’s α coefficient**
T11 Have clear group learning objectives	0.504	0.963	0.961
T12 Number of times members post and communicate in the discussion area	0.707	0.96	
T13 Members are willing to share learning resources	0.803	0.958	
T14 Members’ awareness and compliance with group norms	0.73	0.96	
T15 Members have a sense of collective honor	0.834	0.958	
T21 Members have clear learning plans	0.802	0.958	
T22 How often members visit the online learning platform	0.707	0.96	
T23 The duration of a member’s single visit to the online learning platform	0.659	0.961	
T24 Number of times members download online resources	0.663	0.961	
T25 Ability to collect and screen information and build knowledge	0.855	0.957	
T26 Experience summary and condensation in the learning process	0.88	0.957	
T31 Reasonable division of labor within the community	0.847	0.957	
T32 Members are clear about their professional development orientation	0.816	0.958	
T33 Coverage of online resources	0.825	0.958	
T34 The exemplary role of typical figures in the community	0.778	0.959	
T35 The guiding ability and interactive efficiency of professional teachers	0.812	0.958	
T36 The supervision of the teaching process by the university and college supervisors	0.75	0.934	

**Table 5:** Validity analysis.

**Index name**	**Factor load factor**			**Commonality**
				**(Common factor variance)**
	Factor 1	Factor 2	Factor 3	
T11 Have clear group learning objectives	0.878	0.268	0.135	0.861
T12 Number of times members post and communicate in the discussion area	0.14	0.527	0.504	0.552
T13 Members are willing to share learning resources	0.415	0.467	0.248	0.705
T14 Members’ awareness and compliance with group norms	0.368	0.825	0.222	0.865
T15 Members have a sense of collective honor	0.252	0.79	0.366	0.821
T21 Members have clear learning plans	0.743	0.29	0.339	0.751
T22 How often members visit the online learning platform	0.605	0.55	0.307	0.763
T23 The duration of a member’s single visit to the online learning platform	0.889	0.23	0.183	0.877
T24 Number of times members download online resources	0.866	0.147	0.219	0.82
T25 Ability to collect and screen information and build knowledge	0.821	0.322	0.184	0.811
T26 Experience summary and condensation in the learning process	0.886	0.271	0.21	0.903
T31 Reasonable division of labor within the community	0.854	0.346	0.221	0.897
T32 Members are clear about their professional development orientation	0.288	0.304	0.818	0.844
T33 Coverage of online resources	0.274	0.288	0.832	0.851
T34 The exemplary role of typical figures in the community	0.424	0.256	0.664	0.837
T35 The guiding ability and interactive efficiency of professional teachers	0.287	0.379	0.586	0.901
T36 The supervision of the teaching process by the university and college supervisors	0.248	0.744	0.234	0.429
Characteristic root value (before rotation)	10.104	1.962	0.991	–
Variance interpretation rate% (before rotation)	63.15%	12.26%	6.20%	–
Cumulative variance interpretation rate% (before rotation)	63.15%	75.42%	81.61%	–
Characteristic root value (after rotation)	6.066	3.838	3.155	–
Variance interpretation rate% (after rotation)	37.91%	23.99%	19.72%	–
Cumulative variance interpretation rate% (after rotation)	37.91%	61.89%	81.61%	–
KMO value	0.912			–
Bart sphere value	1076.284			–
*df*	120			–
*p* 值	0			–

### 4.3. Confirmatory factor analysis

The questionnaire was analyzed by confirmatory factor analysis. There were 3 dimensions and 16 indicators in total. The effective sample size was 247, which was 10 times larger than the analysis quantity. This was in line with the analysis premise. (1) Aggregation validity analysis: the SPSS analysis software outputs the results, and the average variance extraction AVE values of the three key dimensions are 0.678, 0.613, and 0.834, all greater than 0.5, and the CR values are 0.98, 0.925, and 0.952, all greater than 0.7, showing good aggregation validity. (2) Differentiation validity analysis: analyze the discrimination of the three key dimensions of the questionnaire to verify whether there is *a* correlation between the dimensions. The square roots of AVE of each dimension are 0.824, 0.783, and 0.913, with the minimum value >0.765 (the maximum value of the correlation coefficient between factors), indicating that the questionnaire data can be well distinguished. (3) Common method deviation analysis: from the calculation results of model fitting indicators, except that NNFI is close to the recommended value, other indicators (as shown in Table 6) meet the requirements of the recommended value, indicating that the model fitting has a good result.

**Table 6:** Model fitting indicators.

**Indicator items**	***p***	***χ***^**2**^**/df**	**GFI**	**RMSEA**	**RMR**	**CFI**	**NFI**	**NNFI**
Measured value	0.06	2.118	0.902	0.088	0.047	0.915	0.942	0.875
recommended value	>0.05	<3	>0.9	<0.10	<0.05	>0.9	>0.9	>0.9

### 4.4. Analysis results of influencing factors based on the analytic hierarchy process

The analytic hierarchy process (AHP) is used to analyze the key factors. First, the analytic hierarchy process model and the third-order judgment matrix are formed. The importance of each level to the previous level is calculated through the weighted average by consulting the literature and educational experts (see Tables 7–10). Query the random RI table according to the obtained data. The RI values and CR values of items in the first and second layers are 0.52, 0.89, 1.41, and 0.89 and 0, 0.001, 0.013, and 0.062 (CR values <0.1), respectively, indicating that the consistency test of the judgment matrix has passed, and the final weight and hierarchical ranking results have been obtained, as shown in Table 11.

**Table 7:** Scores of importance.

Index	A1	A2	A3
A1	/	D1,2	D1,3
A2	/	/	D2,3
A3	/	/	/

**Table 8:** AHP analysis results.

**Term**	**Feature vector**	**Weight value**	**Maximum characteristic value**	**CI value**
Group solidarity	0.857	28.57%	3	0
Group development	1.714	57.14%		
Group support	0.429	14.29%		

**Table 9:** Random consistency test of the first layer.

**Maximum characteristic root**	**CI value**	**RI value**	**CR value**	**Consistency inspection results**
3	0	0.52	0	Adopt

**Table 10:** Random consistency test of the second layer.

**Maximum characteristic root**	**CI value**	**RI value**	**CR value**	**Consistency inspection results**
4.001	0	0.89	0.001	Adopt
8.125	0.018	1.41	0.013	Adopt
4.185	0	0.89	0.062	Adopt

**Table 11:** Weights of key dimensions.

**Criterion Layer**	**Weight 1**	**Indicator layer**	**Weight 2**	**Weight 3**	**sorting**
Group solidarity	0.286	Number of times members post and communicate in the discussion area	0.220	0.063	8
		Members are willing to share learning resources	0.187	0.053	13
		Members’ awareness and compliance with group norms	0.256	0.073	4
		Members have a sense of collective honor	0.337	0.096	2
Group development	0.571	Have clear group learning objectives	0.132	0.075	5
		Members have clear learning plans	0.110	0.063	9
		Reasonable division of labor within the community	0.104	0.059	12
		How often members visit the online learning platform	0.121	0.069	7
		The duration of a member’s single visit to the online learning platform	0.111	0.070	6
		Number of times members download online resources	0.109	0.062	10
		Ability to collect and screen information and build knowledge	0.107	0.061	11
		Experience summary and condensation in the learning process	0.206	0.118	1
Group support	0.143	Members are clear about their professional development orientation	0.116	0.017	16
		Coverage of online resources	0.217	0.031	14
		The exemplary role of typical figures in the community	0.124	0.018	15
		The guiding ability and interactive efficiency of professional teachers	0.543	0.078	3

## 5. Conclusion and policy implications

### 5.1. Conclusion

In the context of the Internet of Things (IoT), we try to build up a new online learning community model: (1) First, we introduce the Kolb learning style theory to identify different online learning styles; (2) Second, we use *a* clustering algorithm to identify the nature of different learning style groups; and (3) Third, we introduce the group dynamics theory to design the dimensions of the questionnaire, and we combine the analytic hierarchy process (AHP) method to identify the key influencing factors of *the* online learning community.

The main findings include: (1) “Experience summary and condensation in the learning process” has the highest weight, which indicates that although *an* online learning community is a new way of learning, in the process of online learning of individual members, they keep their daily learning habits, think about the knowledge they have learned, explore the essence through learning phenomena, deeply understand the connection and difference of knowledge points, and then summarize. The process of drawing general conclusions is essential. (2) “Members have a collective sense of honor” ranked second. The collective sense of honor will encourage members to love and care about the community, consciously fulfill their learning obligations, and hold the belief of making contributions and striving for honor. (3) “The guidance ability and interaction efficiency of professional course teachers” ranked third. The online learning community provides learners with a broad learning space and a platform for independent communication. How to guide, organize, strengthen interaction, deepen cognition and achieve goals is a very important responsibility and *a* key role for teachers. (4) “Members’ understanding and compliance with group norms” ranked fourth. In the process of interaction within the learning community, members are directly affected by individual attitudes, subjective norms, and *a* sense of fairness. When individual members actively comply with group norms, they can create a good interactive atmosphere and order to ensure that collective learning activities are completed on time, with quality and quantity guaranteed. (5) “Have a clear group learning goal” ranked fifth. The group has a good productive and functional nature, formulate a clear group goal, and clearly pass it on to internal members so that their thoughts and behaviors are consistent. It can improve the group’s ability to act and create value together, and enhance members’ sense of identity and belonging.

In addition, indicators with low weight value include “members are willing to share resources,” “coverage of online resources,” “exemplary role of typical figures in the community,” “members are clear about their professional development positioning,” etc. The participants of the first two indicators have been relatively satisfied with the convenient network. There are various types of typical characters, which are easy to be inconsistent with online courses. However, as students, they usually pay little attention to the macro definition of professional development orientation and related issues and have little consideration. They cannot clarify the close relationship between themselves and the training direction, professional orientation, professional courses, etc.

### 5.2. Discussion

The main contribution of this *article* is summarized as follows:

1. We introduce Kolb learning style theory to identify different online learning styles, and on this basis, we use *a* clustering algorithm to identify and distinguish the characteristics of students with different learning styles and establish an online learning community based on different learning styles in the major of business administration, which is completely different from the traditional manual grouping. More conducive to the formation of “inter-group homogeneity, intra-group heterogeneity” team learning situation promote the online learning community learning efficiency improvement.
2. The group dynamics theory is introduced to design the questionnaire dimension, making the establishment of the evaluation index system more scientific and normative, which is completely different from the previous evaluation index selection based on subjective perception.
3. Combined with the analytic hierarchy process (AHP), the common key influencing factors of online learning are identified, and the effects of different influencing factors and possible causes are analyzed, which provides a solid basis for proposing feasible strategies to improve the efficiency of online learning.
4. On the whole, it has formed a complete set of logic and ideas from program design, program implementation, effect test, and improvement strategy, which is different from the previous single-level research on the improvement of teaching methods and teaching strategy.

Admittedly, this article has two limitations. First, due to the limitation of the survey sample, this study could not obtain more extensive research data, so the accuracy of the conclusion in a wider range could not be verified. Second, from the perspective of the construction and development of the online community, this *article* discusses as comprehensively as possible the factors that influence the improvement of the efficiency of online learning in universities during the COVID-19 pandemic, but there is still room for further improvement of the integrity of the indicators. Therefore, in future research, we can further improve the data and establish a dynamic supplementary database. At the same time, a more diversified index system and analytical framework can be constructed. On this basis, the influencing factors, driving mechanism, and continuous improvement strategy of the development of *the* online learning community can be further analyzed.

### 5.3. Policy implications

1. Reviewing the online learning process and clarifying the “causes and consequences.” Reviewing is a means to promote personal knowledge accumulation and ability improvement through reflection and summary of past experiences and lessons. Compared with the traditional offline education model, the online platform is more open and free, and the control of the learning process affects the overall efficiency of online learning. Learners should change their thinking, master and use the characteristics of the online platform’s efficiency and convenience, do a good job in the pre*-*evaluation, target control, achievement and target comparison of the entire learning process, and find out the problems in knowledge acquisition, knowledge understanding, knowledge application, knowledge expansion, etc. Regularly summarize professional knowledge, sort out the knowledge framework, sort out the knowledge levels, excavate the commonalities and differences in knowledge points, and form a complete knowledge system and structure.
2. Strengthen the collective concept and enhance the sense of honor of *the* online learning community. The sense of collective honor encourages group members to maintain a positive attitude and encourages the morale of learners. To cultivate *a* collective sense of honor, first of all, we should strengthen the community awareness of community members and strengthen the correlation between individual members and the community. Teachers should provide rich teaching activities and diversified mixed teaching methods, enhance learners’ interest in learning, improve individual members’ participation in the classroom, and thus strengthen their recognition of the community. The community should establish a harmonious atmosphere of interpersonal communication, guide community members to communicate on an equal footing and cooperate in a friendly manner, and enhance mutual friendship and improve *the* collective centripetal force. Second, set up stage learning objectives oriented to the completion of independent learning tasks, form a competition system and competition system among communities, and implement the combination of individual incentive and group incentive*s*, so that individual members can fully experience *a* sense of achievement and honor and accelerate the integration of the organization.
3. Clarify the orientation of teachers and strengthen the role of guidance. The online learning community creates a convenient way for multidirectional communication and forms a network interactive structure. Teachers not only transfer knowledge to learners but also play the role of learner assistant*s*. The guiding role of teachers is reflected in two aspects. The process management of *the* online classroom*s* and the effective organization of *the* online learning communit*ies* are the basis. The multilevel and multiangle communication between “decentralized” individual members can give full play to learners’ autonomy and initiative, and inject impetus into their long-term development. It is the responsibility to cultivate inspiring thinking and a lively cultural atmosphere. By relying on professional knowledge, skills, and emotional attitudes, we can carry out *an* equal, free, and effective dialog between teachers and students, which can fully stimulate the vitality and vitality of online classes.
4. Formulate group norms and improve the evaluation and assessment mechanism. Strengthen the interactive atmosphere of the online learning community and ensure the sustainable development of the community through effective institutional norms and the parallel way of assessment and evaluation. On the one hand, it is necessary to formulate practical community norms and control constraints; on the other hand, it is necessary to establish and improve the evaluation and assessment mechanism. Perfect community norms can give full play to learners’ initiative and consciousness and provide a good atmosphere for interaction between individuals. At the same time, evaluation is carried out for each stage of learning, forming diagnostic evaluation analysis at the beginning of learning, motivational evaluation analysis at the process stage, and summary evaluation at the end to ensure the frequency and quality of online learning. Individual members who violate group norms should be punished to maintain the authority of the norms. Through institutional enforcement and flexible surveys, we should create a good style of study, promote the community members to interact and communicate equally and independently and achieve the long-term development of the community.
5. Clear community goals and enhance *the* positive drive. In the online learning community, members have different expectations for the results of online learning and set different personal goals. Group goals help members to have a deeper understanding of the community. They should follow the important principles of overall, clarity, measurability, accessibility, etc. to achieve the driving role of individual members. Once the group goal is established, individuals should form corresponding individual goals according to the group goal and form a clear and staged plan for the online learning process. The realization of the overall goal depends on the completion of the individual goal, and the individual goal cannot be separated from the positive guidance of the overall goal. Coordinating the goal relationship between individuals and groups, and playing a two-way role in promoting the formation and sustainable development of the online learning community is the core force.

## Data availability statement

The original contributions presented in the study are included in the article/supplementary material, further inquiries can be directed to the corresponding author.

## Author contributions

XL: first draft writing. ZP: review writing. All authors contributed to the article and approved the submitted version.

## Funding

This research was funded by Anhui Province Quality Engineering Project: Curriculum Ideological and Political Demonstration Course (2020szsfkc0324); First-class Courses (2021xxkc031); Quality Engineering Project of Anhui Science and Technology University: Research on the Intelligent Construction of “Online Learning Community” for professional courses in colleges and universities in the post-epidemic Period - A case study of Business Administration major (x202012).

## Conflict of interest

The authors declare that the research was conducted in the absence of any commercial or financial relationships that could be construed as a potential conflict of interest.

## Publisher’s note

All claims expressed in this article are solely those of the authors and do not necessarily represent those of their affiliated organizations, or those of the publisher, the editors and the reviewers. Any product that may be evaluated in this article, or claim that may be made by its manufacturer, is not guaranteed or endorsed by the publisher.
